# Chloroquine, an Anti-Malaria Drug as Effective Prevention for Hantavirus Infections

**DOI:** 10.3389/fcimb.2021.580532

**Published:** 2021-03-15

**Authors:** Valentijn Vergote, Lies Laenen, Raf Mols, Patrick Augustijns, Marc Van Ranst, Piet Maes

**Affiliations:** ^1^ Laboratory of Clinical Virology, Zoonotic Infectious Diseases Unit, Department of Microbiology, Immunology and Transplantation, Rega Institute, KU Leuven, Leuven, Belgium; ^2^ Department of Pharmaceutical and Pharmacological Sciences, Drug Delivery and Disposition, KU Leuven, Leuven, Belgium

**Keywords:** hantavirus, Andes virus, prophylactic treatment, osmotic pump, Syrian hamster animal model, chloroquine (CQ), antiviral therapies

## Abstract

We investigated whether chloroquine can prevent hantavirus infection and disease *in vitro* and *in vivo*, using the Hantaan virus newborn C57BL/6 mice model and the Syrian hamster model for Andes virus. *In vitro* antiviral experiments were performed using Vero E6 cells, and Old World and New World hantavirus species. Hantavirus RNA was detected using quantitative RT-PCR. For all hantavirus species tested, results indicate that the IC_50_ of chloroquine (mean 10.2 ± 1.43 μM) is significantly lower than the CC_50_ (mean 260 ± 2.52 μM) yielding an overall selectivity index of 25.5. We also investigated the potential of chloroquine to prevent death in newborn mice after Hantaan virus infection and its antiviral effect in the hantavirus Syrian hamster model. For this purpose, C57Bl/6 mother mice were treated subcutaneously with daily doses of chloroquine. Subsequently, 1-day-old suckling mice were inoculated intracerebrally with 5 x 10^2^ Hantaan virus particles. In litters of untreated mothers, none of the pups survived challenge. The highest survival rate (72.7% of pups) was found when mother mice were administered a concentration of 10 mg/kg chloroquine. Survival rates declined in a dose-dependent manner, with 47.6% survival when treated with 5 mg/kg chloroquine, and 4.2% when treated with 1 mg/kg chloroquine. Assessing the antiviral therapeutic and prophylactic effect of chloroquine in the Syrian hamster model was done using two different administration routes (intraperitoneally and subcutaneously using an osmotic pump system). Evaluating the prophylactic effect, a delay in onset of disease was noted and for the osmotic pump, 60% survival was observed. Our results show that chloroquine can be highly effective against Hantaan virus infection in newborn mice and against Andes virus in Syrian hamsters.

## Introduction

Hantaviruses are enveloped, negative-sense single stranded RNA viruses belonging to the family *Hantaviridae* (Order *Bunyavirales).* The hantaviral genome consists of a small (S), medium (M), and large (L) segment encoding a nucleocapsid protein, two surface glycoproteins (Gn and Gc) and an RNA-dependent RNA polymerase, respectively (Maes et al., 2004). Several hantaviruses (e.g. Puumala virus, Tula virus, Sin Nombre virus, Andes virus) have an additional overlapping (+1) open reading frame on their S segment, coding for a nonstructural protein with a putative inhibitory role in viral replication by acting as an interferon antagonist (Meissner et al., 2002; Jaaskelainen et al., 2007; Virtanen et al., 2010; Vera-Otarola et al., 2012). Each hantavirus species has a single (or a few closely related) rodent or insectivore species as its natural host, and recently also fish and reptiles were identified as possible hosts (Shi et al., 2018). These reservoir hosts are presumably chronically infected without manifesting apparent signs of disease. Upon human transmission, two clinical hantavirus diseases can arise: hantavirus pulmonary syndrome (HPS) and hemorrhagic fever with renal syndrome (HFRS). Both disorders are associated with abrupt fever, malaise and other flu-like symptoms, acute thrombocytopenia, increased vascular permeability, and renal and/or pulmonary dysfunction. Hantaviruses from the Old World (Europe and Asia), with Hantaan virus as prototype, cause HFRS with the kidney as primary target; viruses from the New World (North and South-America), with Sin Nombre virus as prototype, mainly target the lungs, causing HPS.

The main treatment of severe HPS or HFRS cases is purely supportive, mostly in intensive care unit surroundings. This involves mechanical ventilation or even extra-corporeal membrane oxygenation for HPS and all forms of extra-corporeal blood purification (mostly hemodialysis) for HFRS (Clement et al., 2006). Although hantaviruses are found across the globe, little has been published concerning antivirals being tested in hantavirus disease or infection models (Jonsson et al., 2008). The most recently described antiviral that was potent *in vitro* as well as in the *in vivo* lethal disease model for Andes virus was favipiravir, also known as T-705 (Safronetz et al., 2013). T-705 was shown to be effective by decreasing viral RNA and infectious titers, and exhibited significant higher survival rates in the lethal hamster model. The only licensed antiviral well described for hantaviruses is ribavirin (McKee et al., 1988; Huggins et al., 1991). Ribavirin, a broad-spectrum inhibitor of RNA virus replication (Severson et al., 2003), is the only licensed antiviral treatment or prevention available that acts on the primary viral etiology of HFRS or HPS. However, ribavirin is not widely available and should be given only intravenously and early in the clinical course (Chapman et al., 1999). This implies that the use of ribavirin intravenously remains limited to quickly recognized and severe forms of disease, where the clinician cannot wait for serological results for his diagnosis and has deemed the potential benefits of this medication to outweigh its non-negligible side-effects.

Besides these antiviral compounds, other products and biologicals are of potential interest as antivirals for use against hantaviruses. BCX4430 is a broadly active nucleoside analog with antiviral activity in filovirus, yellow fever (Julander et al., 2014), and Zika virus infection models (Julander and Siddharthan, 2017). Goose polyclonal antibodies delivered *via* a DNA vaccine was shown to be highly effective in the Andes hamster model (Haese et al., 2015; Gowen and Hickerson, 2017).

Here we describe chloroquine, a four-aminoquinoline, which emerged during the first part of the 20th century as an effective quinine substitute and is still a drug of choice against malaria (Rolain et al., 2007). Chloroquine is known to elicit antiviral effects *in vitro* against several viruses including hepatitis C virus (Blanchard et al., 2006), HIV type 1 virus (Savarino et al., 2001), influenza virus A and B (Ooi et al., 2006), SARS coronavirus (Keyaerts et al., 2004; Vincent et al., 2005), Chikungunya virus (Khan et al., 2010), and others (Rolain et al., 2007). A randomized, double-blind, placebo-controlled trial testing chloroquine for the prevention of influenza showed no antiviral effect (Paton et al., 2011). For HIV-1, the *in vivo* potential of hydroxychloroquine has been studied in comparison to zidovudine, a known antiretroviral product active against HIV showing promising results (Sperber et al., 1997). At the moment of writing, chloroquine is of key interest around the world in light of the Sars-CoV-2 pandemic (Cortegiani et al., 2020; Liu et al., 2020; Moore, 2020).

The main mechanism of action is based on the interaction of chloroquine with endosomal mediated virus entry. Chloroquine increases the pH in endosomes, prohibiting viral release by trapping the virus. Moreover, chloroquine is believed to have immunomodulatory capacities (Jang et al., 2006). In this study, we evaluated chloroquine for its antiviral potential against the hantavirus species Dobrava-Belgrade virus, Hantaan virus, and Sin Nombre virus *in vitro* and against Hantaan virus *in vivo* in 1-day-old C57Bl/6 mice. Additionally, we evaluated the effect of chloroquine on Andes virus *in vivo* in the Syrian hamster model.

## Materials and Methods

### Ethics Statement

This study was approved and supervised by the KU Leuven Animal Welfare Body (approval LA1210186) in compliance with Belgian and European statutes and regulations relating to animals and experiments involving animals. All animal experiments were carried out in a BSL-3+ animal facility.

### Virus and Cell Culture

The virus strains used in this study were Dobrava-Belgrade virus (DOBV) strain SK/Aa (Klempa et al., 2005), Hantaan virus (HTNV) strain 76-118 (Lee et al., 1978), Sin Nombre virus (SNV) strain NMR11 (Chizhikov et al., 1995), and Andes virus strain Chile-9717869. Vero E6 cells (American Type Culture Collection, C1008) were cultured in minimum essential medium (DMEM) supplemented with 10% heat-inactivated fetal calf serum. The maintenance medium for virus propagation was identical but contained 2% fetal calf serum. All hantaviruses used in this study were propagated for 10 days on monolayers of Vero E6 cells. Cells and viruses were cultured in a humified environment at 37°C supplemented with 5% CO_2_.

### Hantavirus Quantitation by Using Quantitative RT-PCR

qRT-PCR was carried out using the Eurogentec One Step RT qPCR kit (Eurogentec, Seraing, Belgium) with the ABI Prism 7500 Fast Sequence Detection System (Applied Biosystems) as described previously (Maes et al., 2007). Briefly, the reaction was conducted in a 25 μl volume containing 5 μl of extracted RNA, 12.5 μl of One step RT qPCR MasterMix (Eurogentec, Seraing, Belgium) containing ROX as a passive reference, 900 nM forward and reverse primer, 250 nM probe, which was labeled at the 5’ end with the fluorescent dye six-carboxyfluorescein (FAM) as the reporter dye and at the 3’ end with the quencher dye six-carboxytetramethylrhodamine (TAMRA), and 0.125 μl Euroscript/RNase inhibitor (Eurogentec). Reverse transcription was initiated at 48°C for 30 min, followed by PCR activation at 95°C for 10 min and 45 cycles of a two-step incubation at 95°C for 15 s (denaturation) and 60°C for 1 min (primer annealing and elongation). The reporter dye (FAM) signal was measured against the internal reference dye (ROX) to normalize for non-PCR-related fluorescence emissions. The threshold cycle (CT) was defined as the fractional cycle number at which the reporter fluorescence, generated by cleavage of the probe, reached a threshold defined as 10 times the standard deviation of the mean baseline emission. In order to allow absolute hantavirus quantitation, cRNA standards were constructed and used for the generation of a standard curve, as described previously (Maes et al., 2009). Primer and probe sequences are summarized in **Table 1** .

**Table 1:** Primer and TaqMan probe sequences.

	Forward primer (5’ to 3’)	Reverse primer (5’ to 3’)	TaqMan probe (5’ to 3’)
**DOBV**	TCCCGTGCAAGCTACTATCTGA	GCGCTCCTTGTCTTTGATTCA	ACCAAAGGCCCATCCACCAATCGT
**HTNV**	CTGGATTTAAACCATTTGGATATTGA	TATCGGGACGACAAAGGATGTA	AGCAGACTGGCTGAGCATCATCGTCTATCT
**SNV**	TACTCCTCATTCAGTTTGGGTCTTT	TCCCGGCCACATA TAATGCT	CATGTGCTCCAGATCGTTGTCCACCT

### Compound and Antiviral Assay

We tested chloroquine phosphate (7-chloro-4-[[4-(diethylamino)-1-methylbutyl]amino] quinoline phosphate, Alpha pharma, Braine-l’Alleud, Belgium). All concentrations used are chloroquine concentrations.

Antiviral measurements were based on the reduction of hantavirus titer on Vero E6 cells. Monolayers of Vero E6 cells were infected with 10^4^ hantavirus copies per ml in the presence of various concentrations of chloroquine (chloroquine, ranging from 0.032 to 500 µM). After 5 days of incubation at 37°C 5% CO_2_, cell supernatants were collected and the viral titer was determined by using the qRT-PCR described above.

### Cytotoxicity Assay

Cytotoxicity measurements were based on the viability of Vero E6 cells in the presence of various concentrations of chloroquine as previously described (Keyaerts et al., 2004). After a 5-day incubation period, the number of viable cells was quantified by a tetrazolium-based colorimetric method, in which the reduction of MTS (CellTiter 96 AQueous One Solution kit, Promega, Netherlands) by mitochondrial dehydrogenases to a soluble colored formazan was measured in a spectrophotometer at 492 nm. The cytotoxic concentration was defined as the concentration of the compound that reduced cell viability by 50% (50% cytotoxic concentration, CC_50_).

### *In Vivo* Evaluation of Chloroquine in 1-Day-Old C57Bl/6 Pups

Six-week-old male and female C57Bl/6 mice were obtained from Janvier Labs (Le Genest-Saint-Isle, France). Pregnant mice were treated daily with several dilutions of chloroquine subcutaneously (1, 5, and 10 mg/kg), starting with the treatment 2 days before birth of the pups. Within 24 h of birth, neonatal C57Bl/6 mice were inoculated intracerebrally with 5 x 10^2^ Hantaan virus particles. The surviving mice were counted for up to 60 days after the infection.

### *In Vivo* Evaluation of Chloroquine in the Syrian Hamster Model for Andes Virus

For the *in vivo* testing of chloroquine, we used the animal disease model described by Hooper et al, which was later recreated in our lab and adapted to other hantavirus strains (Hooper et al., 2001; Vergote et al., 2017). In short, upon infection with Andes virus, golden Syrian hamsters develop human-like disease symptoms with 100% mortality at an average of 14 days post infection (dpi).

Five-week-old female Syrian hamsters were obtained from Janvier Labs (Le Genest-Saint-Isle, France). Animals received intraperitoneal injections (IP) of chloroquine (60 mg/kg/day; 90 mg/kg/day and two times 90 mg/kg/day) up to 8 dpi or subcutaneously (SC) delivered chloroquine (100 mg/kg/day), depending on the treatment regime that was tested. An ALZET osmotic pump (Charles River Benelux) was used for subcutaneous chloroquine administration during 14 days at a rate of 5.0 µl/h. Chloroquine treatment was initiated 1 day before ANDV challenge for both the IP and the prophylactic SC group. For the therapeutic SC group chloroquine administration was started 5 dpi. A schematic overview is available in **Supplementary Figure 1**. On day 0, all animals received 200 PFU of ANDV intramuscularly (Hooper et al., 2001).

### *In Vivo* Concentration of Subcutaneously Delivered Chloroquine in Syrian Hamsters

Two groups of five Syrian hamsters were subcutaneously treated with 100 mg/kg chloroquine using the Alzet osmotic pump and followed for 17 days. To comply with animal regulations, blood withdrawal was performed using a specific scheme. Group 1 was bled on days 1, 4, 9, 14, and 17. Group 2 was bled on days 2, 7, 11, and 15. Chloroquine, bisdesethylchloroquine, and desethylchloroquine levels in the blood were determined using HPLC as described elsewhere (Augustijns and Verbeke, 1990).

## Results

### *In Vitro* Evaluation of Chloroquine in Vero E6 Cells

The *in vitro* activity of chloroquine was tested on Vero E6 cells. Cytotoxicity in these cells was measured in parallel with the antiviral activity. These experiments, done in triplicate, were repeated three times. In the virus yield assays, where viral RNA was quantified using qRT-PCR 7 dpi, the IC_50_ was 9.51 μM, 10.49 μM, and 10.47 μM for respectively Dobrava-Belgrade virus, Hantaan virus, and Sin Nombre virus. In **Supplementary Figure 2**, virus inhibition and cell inhibition for the different tested concentrations of chloroquine for the different hantaviruses are visualized. The CC50 for chloroquine was 260 µM, giving a selectivity index (SI) of 27.3 for Dobrava-Belgrade virus and 24.8 for Hantaan and Sin Nombre virus (**Table 2**).

**Table 2:** Overview of the antiviral activity of chloroquine against Dobrava-Belgrade virus, Hantaan virus, and Sin Nombre virus with their 50% inhibitory concentration (IC_50_), 50% cytotoxic concentration (CC_50_), and selectivity index (SI).

	IC_50_ (µM)	CC_50_ (µM)	SI
**Dobrava-Belgrade virus**	9.51 ± 1.1	260 ± 2.52	27.3
**Hantaan virus**	10.49 ± 1.8	260 ± 2.52	24.8
**Sin Nombre virus**	10.47 ± 1.4	260 ± 2.52	24.8

### *In Vivo* Evaluation of Chloroquine in 1-Day-Old C57Bl/6 Pups

**Figure 1:**
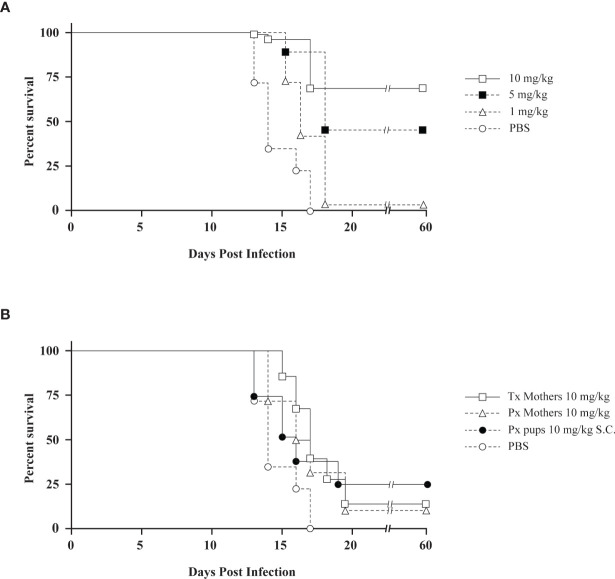
**(A)** The preventive effect of chloroquine on Hantaan virus infection in newborn mice depicted by Kaplan-Meier survival plots. Mothers were treated daily with different concentrations of chloroquine for 16 days. Treatment started 2 days before birth. (○) PBS; (▵) 1 mg/kg chloroquine, (▪) 5 mg/kg chloroquine, (□) 10 mg/kg chloroquine. **(B)** The therapeutic effect of chloroquine on Hantaan virus infection in newborn mice. Symbols: (○) PBS; (□) mothers treated with daily doses of 10 mg/kg chloroquine started after infection (16.7% survival); (▵) mothers treated with 10 mg/kg chloroquine before birth of the pups (days -2 and -1, 12.5% survival); (●) pups treated daily with 10 mg/kg chloroquine subcutaneously (25% survival). Tx, therapeutic treatment; Px, prophylactic treatment.

We also investigated whether chloroquine could prevent death in newborn C57Bl/6 mice as a result of infection with Hantaan virus. Hantaan virus and Seoul virus infections of suckling C57Bl/6 mice have been reported to be lethal, albeit without reflecting the symptoms seen in humans (Yoo et al., 1993). The primary target of infection is the same in newborn C57Bl/6 mice as in humans, namely the capillary endothelium (Kurata et al., 1983). In the *in vivo* experiments, C57Bl/6 mother mice were treated by subcutaneous injection with daily doses of chloroquine, which is, due to its physicochemical characteristics as a lipophilic weak base, excreted in the mother’s milk (Keyaerts et al., 2009). Treatment was started in these mother mice 2 days before birth and stopped 16 days later. The mice were followed for 60 days. Subsequently, 1-day-old suckling mice were inoculated intracerebrally with 5 x 10^2^ Hantaan virus particles. In litters of untreated mothers, none of the pups survived virus challenge. The highest survival rate (16 out of 22 pups, 72.7%) of the pups was found when mother mice were treated with daily doses of 10 mg/kg chloroquine. Survival rates declined in a dose-dependent manner, with 47.6% survival when treated with 5 mg/kg chloroquine (10 out of 21 pups), and 4.2% survival (one out of 24 pups) when treated with 1 mg/kg chloroquine (**Figure 1A**). Daily doses of chloroquine greater than 10 mg/kg resulted in death of the offspring. Moreover, we also investigated the therapeutic effect of chloroquine. If treatment with 10 mg/kg chloroquine of C57Bl/6 mother mice was started directly after infection, the litters showed 16.7% survival (three out of 18 pups, **Figure 1B**). Furthermore, if mothers were treated only before birth of the pups (treatment with chloroquine was stopped before infection), survival of the pups was 12.5% (two out of 16 pups, **Figure 1B**). Daily treatment of 1-day-old suckling mice from untreated mothers with subcutaneous injection of chloroquine induced only partial protection (three out of 12 pups survived, 25%). Doses of chloroquine higher than 7 mg/kg given subcutaneously to suckling C57Bl/6 mice were found to be lethal.

### *In Vivo* Evaluation of Chloroquine in the Syrian Hamster Model for Andes Virus

#### Intraperitoneal Injection

Four groups of five hamsters were challenged with 200 PFU of ANDV (IM) on day 0. Three groups received a first dosage of chloroquine 1 day prior to infection, the control group did not receive any treatment and were solely challenged with ANDV on day 0. A first group received 60 mg/kg/day of chloroquine IP (CQ60), the second group 90 mg/kg/day IP (CQ90), and the third group received two times a day 90 mg/kg IP (CQ90x2). The treatment regime was given up to 8 dpi.

**Figure 2:**
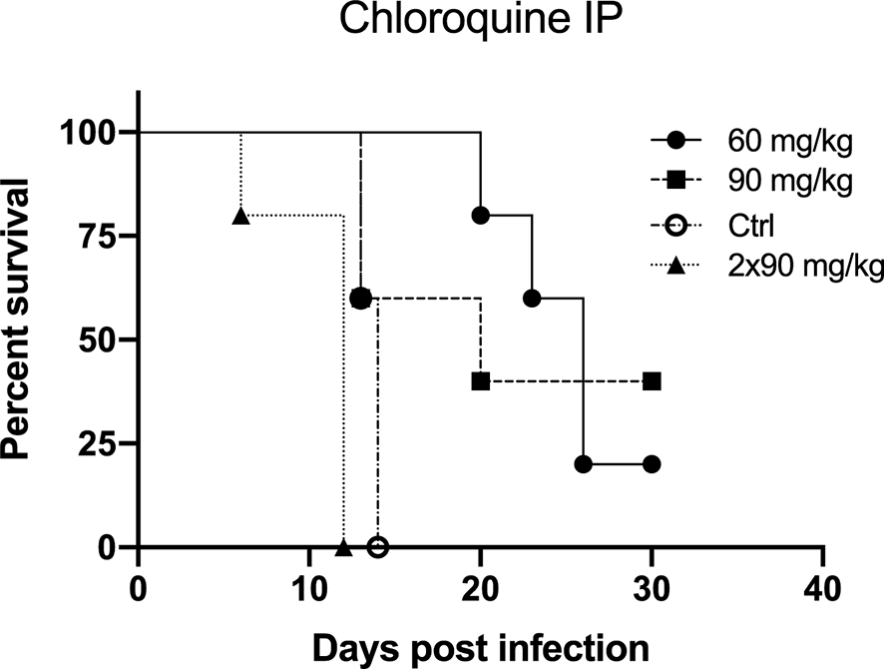
The preventive effect of chloroquine on Andes virus infection in the lethal hamster disease model by intraperitoneal injection (IP) depicted by Kaplan-Meier survival plots. Treatment of the hamsters started 1 day prior to infection. On day 0, all hamsters were infected with 200 PFU Andes virus intramuscularly. Three different treatment regimens were tested. The CQ60 group received 60 mg/kg/day chloroquine IP, the CQ90 group received 90 mg/kg/day chloroquine IP and the CQ90x2 received two times a day 90 mg/kg IP. Each treatment group was compared to the control group, which was challenged with the virus but did not receive any preventive therapy. Each group consisted of five hamsters. The CQ60 group showed a significant difference in survival (P value for significance < 0.05) compared to the control group (Log-rank test, p-value = 0.0031; Gehan-Breslow-Wilcoxon test, p-value = 0.0043). The survival curves of the control group and the CQ90 group do not show a significant difference in survival (Log-rank test, p-value = 0.1558; Gehan-Breslow-Wilcoxon test, p-value = 0.3228). The CQ90x2 group displays a significantly worse survival compared to the control group (Log-rank test, p-value = 0.0035; Gehan-Breslow-Wilcoxon test, p-value = 0.0039).

In **Figure 2**, Kaplan-Meier survival plots are illustrated for each group compared to the control group. The difference in survival between the several groups was statistically calculated using the Log-rank test and the Gehan-Breslow-Wilcoxon test (P value for significance < 0.05). Survival between the control group and the CQ60 group was shown to be statistically different (Log-rank test, p-value = 0.0031; Gehan-Breslow-Wilcoxon test, p-value = 0.0043). The control group and the CQ90 group do not show a significant difference in survival (Log-rank test, p-value = 0.1558; Gehan-Breslow-Wilcoxon test, p-value = 0.3228). The third group, which received two times a day 90 mg/kg chloroquine, shows a significantly worse survival curve compared to the control group (Log-rank test, p-value = 0.0035; Gehan-Breslow-Wilcoxon test, p-value = 0.0039).

#### Osmotic Pump System: Prophylactic Treatment

**Figure 3:**
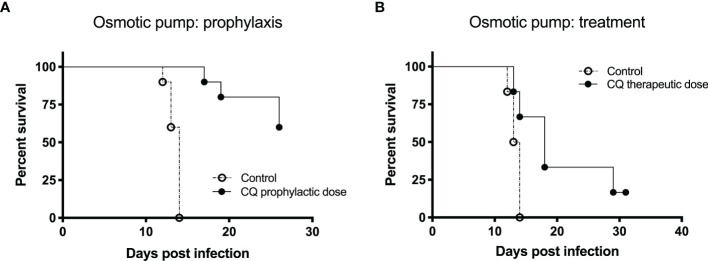
The effect of an osmotic pump system on the prophylactic and therapeutic use of chloroquine for Andes virus infection in hamsters depicted by Kaplan-Meier survival plots. For both experiments, prophylaxis and treatment, 200 PFU of Andes hantavirus was used to challenge the hamsters. The Alzet osmotic pump was used, containing 100 mg/kg chloroquine **(A)** One day prior to infection, the osmotic pump system was implanted subcutaneously to evaluate the prophylactic effect of chloroquine. In both groups, 10 hamsters were used. We see a significant difference (p-value for significance < 0.05) between the survival of the two groups (Log-rank test, p-value < 0.0001; Gehan-Breslow-Wilcoxon test, p-value < 0.0001). All the animals of the control group died within 14 days post infection (dpi). For the treated group, we see a 60% survival 26 dpi. **(B)** Five dpi, the osmotic pump system was implanted subcutaneously to test the therapeutic effect of chloroquine. The Kaplan-Meier survival curves of the two groups (six hamsters each) being displayed show a significant difference in survival (Log-rank test, p-value < 0.0249; Gehan-Breslow-Wilcoxon test, p-value < 0.0361). All hamsters included in the control group died within 14 dpi. Two hamsters of the treated group died on days 13 and 14, respectively. Seventeen dpi, 66% of the animals in the treated group were still alive. On day 31, one hamster was still alive.

In this experiment, two groups of 10 Syrian hamsters were used. The treated group was implanted an osmotic pump containing 100 mg/kg chloroquine subcutaneously 1 day prior to infection (day -1). On day 0 (ANDV challenge), both groups received 200 PFU of Andes virus intramuscularly. As expected from the well-characterized lethal disease model (Hooper et al., 2001), disease and lethality should be induced around 14 dpi. In **Figure 3A**, the Kaplan-Meier survival curves of the control group and the treated group that received the osmotic pump system 1 day prior to infection are visualized. The difference in survival between the two groups was statistically calculated using the Log-rank test (p-value < 0.0001) and the Gehan-Breslow-Wilcoxon test (p-value < 0.0001). The used p-value for significance is < 0.05. All the animals of the control group died within 14 dpi. For the treated group we see a 60% survival 26 dpi.

#### Osmotic Pump System: Therapeutic Treatment

In this experiment, two groups of six Syrian hamsters were used. The treated group was implanted an osmotic pump containing 100 mg/kg chloroquine subcutaneously 5 dpi. On day 0 (ANDV challenge), both groups received 200 PFU of Andes virus intramuscularly. As expected from the well-characterized lethal disease model (Hooper et al., 2001), disease and lethality should be induced around 14 dpi. In **Figure 3B**, Kaplan-Meier survival curves of the two groups are displayed, showing a significant difference in survival (Log-rank test, p-value < 0.0249; Gehan-Breslow-Wilcoxon test, p-value < 0.0361). All hamsters included in the control group died within 14 dpi. Two hamsters of the treated group died 13 and 14 dpi, respectively. At 17 dpi, 66% of the animals in the treated group were still alive. At 31 dpi, only one hamster was still alive.

### *In Vivo* Concentration of Subcutaneously Delivered Chloroquine in Syrian Hamsters

**Figure 4:**
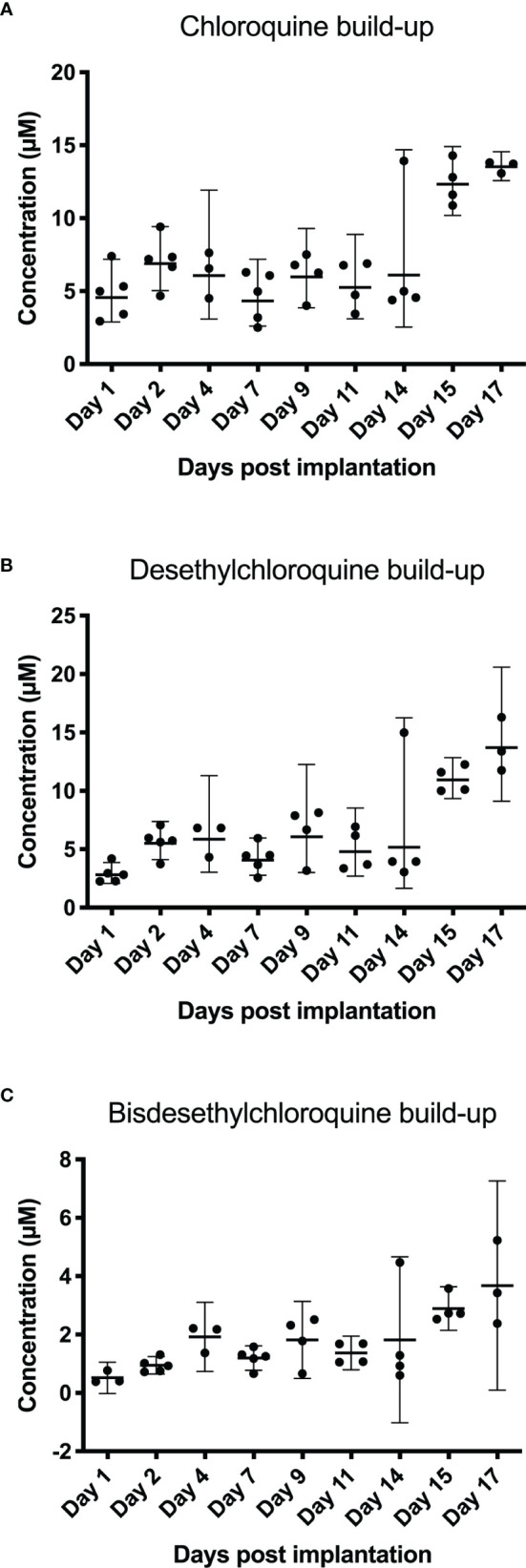
Blood levels after subcutaneous administration of chloroquine using an osmotic pump system. Blood was collected for 17 days post-implantation. Chloroquine, desethylchloroquine, and bisdesethylchloroquine levels were determined using HPLC. In **(A)** chloroquine build-up shows a constant level of chloroquine until day 14. On days 15 and 17, we noticed that blood concentrations were almost three times higher. For chloroquine, the mean concentration is fluctuating between 4.61 and 7.01 µM during the first 14 days, with a mean concentration peak on day 17 of 13.54 µM. The desethylchloroquine levels **(B)** and the bisdesethylchloroquine levels **(C)** in the blood show a similar pattern as chloroquine, as depicted in **(A)** For desethylchloroquine, the mean ranges from 2.90 to 6.48 µM during the first 14 days, with a mean concentration peak on day 17 of 13.82 µM. Bisdesethylchloroquine was fluctuating during the first 14 days between a mean concentration of 0.52 µM and 1.82 µM, leading to a peak on day 17 of 3.68 µM.

Concentrations of chloroquine in the blood, as well as its metabolites, were tested by administering chloroquine to hamsters and analyzing the blood taken on specific days after initiation of the treatment. **Figure 4** shows the different concentrations of chloroquine and its metabolites desethylchloroquine and bisdesethylchloroquine, measured until 17 days post implantation of the chloroquine-containing osmotic pump. From day 1 to day 14, fluctuations in the concentration of chloroquine and its metabolites are observed. For chloroquine, the mean concentration is fluctuating between 4.61 and 7.01 µM during the first 14 days, with a mean concentration peak on day 17 of 13.54 µM. For desethylchloroquine, the mean ranges from 2.90 to 6.48 µM during the first 14 days, with a mean concentration peak on day 17 of 13.82 µM. Bisdesethylchloroquine was fluctuating during the first 14 days between a mean concentration of 0.52 µM and 1.82 µM, leading to a peak on day 17 of 3.68 µM. From day 15 to day 17, higher concentrations were seen for all the analyzed products with concentrations even tripling compared to other days. Data points missing are due to insufficient amounts of blood for analysis.

## Discussion

In our experiments, chloroquine was tested *in vitro* using Vero E6 cells with the Old World hantaviruses Dobrava-Belgrade virus and Hantaan virus, and the New World hantavirus Sin Nombre virus. For all three viruses, the 50% inhibitory concentration (IC50) was significantly lower than the 50% cytotoxic concentration (CC50), with an overall selectivity index of 25.5. No difference in reactivity of chloroquine could be seen between the New World virus Sin Nombre virus and the Old World viruses, Dobrava-Belgrade virus and Hantaan virus. Moreover, including the infection experiments of newborn C57Bl/6 mice with Hantaan virus, it can be concluded that chloroquine can be used in the prevention of infection rather than in the treatment of infection. In **Figures 1A, B**, we clearly distinguished a difference between a prophylactic or therapeutic dosing scheme. This discrepancy, however, might also be due to the use of the specific animal model. Although the primary target of Hantaan virus, the capillary endothelium, is the same in newborn C57Bl/6 mice as in humans, this animal model represents an infection model and not a disease model, since it does not reflect the symptoms seen in humans. A practical animal model for HPS is the Syrian hamster model (Hooper et al., 2001). Andes virus is very lethal in adult Syrian hamsters and the characteristics of the disease in these hamsters, including the incubation period, symptoms of rapidly progressing respiratory distress, and pathologic findings of pulmonary edema and pleural effusion, closely resemble HPS in humans (Wahl-Jensen et al., 2007). For this reason, we tested IP administration of chloroquine as well as an osmotic pump for chloroquine treatment in the Syrian hamster model and noted clear differences in both approaches.

Within the IP chloroquine experiment, we noticed a significant difference in survival between the control group and the 60 mg/kg/day group. We observed mortality in all animals of the control group, with a median survival of 14 days compared to 26 days in the 60 mg/kg/day group. The other two groups, 90 mg/kg/day and the group receiving twice a day 90 mg/kg, showed no significant difference in survival or a significantly worse survival, respectively. We hypothesize that the single and double bulk injection of 90 mg/kg per day of chloroquine have a toxic effect in the animals, leading to a similar or worsened outcome in survival compared to the control group. Based on these findings, we further examined the antiviral potential of chloroquine by using an osmotic pump that acts as a slow release vessel, avoiding a bulk injection. Using this osmotic pump system, we tested the prophylactic and therapeutic use of the drug. We noticed a clear difference in survival when comparing the test groups with the control group for both applications. Using chloroquine in a prophylactic way gave 60% survival 26 dpi. In the control group, all animals died within 14 dpi. The therapeutic use of the drug indicated 66% survival 17 dpi and one animal was still alive 31 dpi, showing a clear delay in death. These results clearly indicate that the use of chloroquine has a potential prophylactic and therapeutic effect against a lethal Andes virus infection. As seen for other viral infections, a combined treatment strategy could be an interesting option against hantavirus infections to reach an elevated antiviral effect. Both therapy efficacy and antiviral resistance are important factors that may be of great importance in a combined therapeutic strategy (Ison, 2017; Flexner, 2018).

Our findings show that the administration route can play an important role in product efficacy. Measured blood chloroquine concentrations were shown to be fluctuating with a concentration increase on day 15 to day 17. Higher blood concentrations starting around 14 days post-implantation can be explained by a concentration build-up due to the slow elimination half-time of chloroquine of 1 to 2 months seen in humans (Nosten and White, 2007). One of the main reasons for this slow elimination is the pH-dependent liposomal entrapment mechanism (Savarino et al., 2003; Hu et al., 2020). Although exact pharmacokinetics of chloroquine for hamsters are not known, the elimination half-time in rats is around 10 days (Mustapha et al., 2015). Ten days is significantly shorter than the half-time measured in humans but still indicates a slow elimination, which could explain the chloroquine build-up. The slow elimination half-time as well as the complex interplay of absorption, distribution and metabolism could explain these fluctuating concentrations as well as the increase starting at day 15. Due to the lipophilic character of chloroquine, the volume of distribution is very high, which might have an effect on chloroquine concentrations over time, leading to the effectiveness of CQ in neutralizing hantavirus.

Using this kind of osmotic pumps not only shows the importance of the method of drug delivery but also the importance of refining animal experiments. The effect of administering drugs *via* a continuous way avoids toxic bulk dose administration. Furthermore, by introducing these osmotic pump systems, stress levels are highly reduced by avoiding daily injections (Grathwohl and Jucker, 2013). Biased intake is avoided when drugs are administered through food and water. All these aspects might have an impact on past and present chloroquine research and antiviral testing in general. Recently, it was shown that chloroquine did not elicit an *in vivo* antiviral effect on Ebola virus (Dowall et al., 2015). In this study, an *in vitro* effect for chloroquine was noticed in MRC-5 cells by reducing EBOV replication. For the *in vivo* experiments, difficulties with the administration route (orally and intravenously) were reported. Another study testing chloroquine showed similar results lacking an *in vivo* effect, leading to their conclusion that a clinical use of chloroquine is unlikely. A study by Porotto and colleagues checking the antiviral effect of chloroquine on Nipah and Hendra virus has proven to elicit an *in vitro* antiviral effect but lacked positive *in vivo* results (Porotto et al., 2009). Our methodology could lead to new insights for the *in vivo* potential of chloroquine as an antiviral agent and more specifically for the use against high-risk viral pathogens and hemorrhagic fever viruses.

One of the major advantages of chloroquine is the long experience of the use of this drug in the treatment of malaria, where it has already demonstrated the safety of short-term administration to humans. For administration in rheumatic diseases, hydroxychloroquine is being preferred due to the lower toxicity risk. (Shukla and Wagle Shukla, 2019). The side effects of chloroquine are mainly related to retinal and cardiac toxicity, which should be considered before treatment. In theory and based on the current knowledge and availability, chloroquine can be used immediately for the prevention of hantavirus infection.

Hantaviruses are spread to humans *via* aerosolized excreta from chronically infected wild rodents. Puumala virus can survive and can be transmitted to other rodents for up to 15 days after being excreted (Kallio et al., 2006) whereas Hantaan virus (HTNV) can remain infectious under optimal conditions for up to 96 days (Hardestam et al., 2007). *Myodes glareolus* or bank voles, the rodent host of Puumala virus, prefers to make their burrows under stacks of wood or near the forest (Van Loock et al., 1999). Therefore, chloroquine, can be of great importance as prophylactic medication for people living in and traveling to hantavirus affected areas (Jonsson et al., 2010). Based on our results, we see a specific setting for chloroquine prophylaxis for people at risk. It is presumed that a strong host immune response upon infection plays a major role in hantaviral pathogenesis by inducing changes in vascular permeability. The immunomodulatory capacities of chloroquine could therefore have a beneficial effect upon treatment.

In conclusion, our results indicate that chloroquine has a strong *in vitro* antiviral activity against several hantavirus species, which include New World as well as Old World viruses. Furthermore, chloroquine was highly effective against Hantaan virus infection in an *in vivo* infection model with 1-day-old suckling mice and showed its prophylactic and therapeutic potential in the lethal Andes virus hamster model. These findings suggest that chloroquine, a ubiquitously available medicine of low cost with little side effects, has a potential new role in the prevention or treatment of hantavirus infections.

## Data Availability Statement

The original contributions presented in the study are included in the article/**Supplementary Material**. Further inquiries can be directed to the corresponding author.

## Ethics Statement

The animal study was reviewed and approved by Ethical Committee for Animal Experimentation.

## Author Contributions

VV and PM conceived and designed the experiments. VV, LL, RM, PA, and PM carried out the experimental work. VV drafted the manuscript. All authors contributed to the article and approved the submitted version.

## Conflict of Interest

The authors declare that the research was conducted in the absence of any commercial or financial relationships that could be construed as a potential conflict of interest.
