# Supplementary figures and images for: Chloroquine, an Anti-Malaria Drug as Effective Prevention for Hantavirus Infections
Source: Front Cell Infect Microbiol. 2021 Mar 15;11:580532. doi: 10.3389/fcimb.2021.580532 (PMC8006394; doi:10.3389/fcimb.2021.580532)

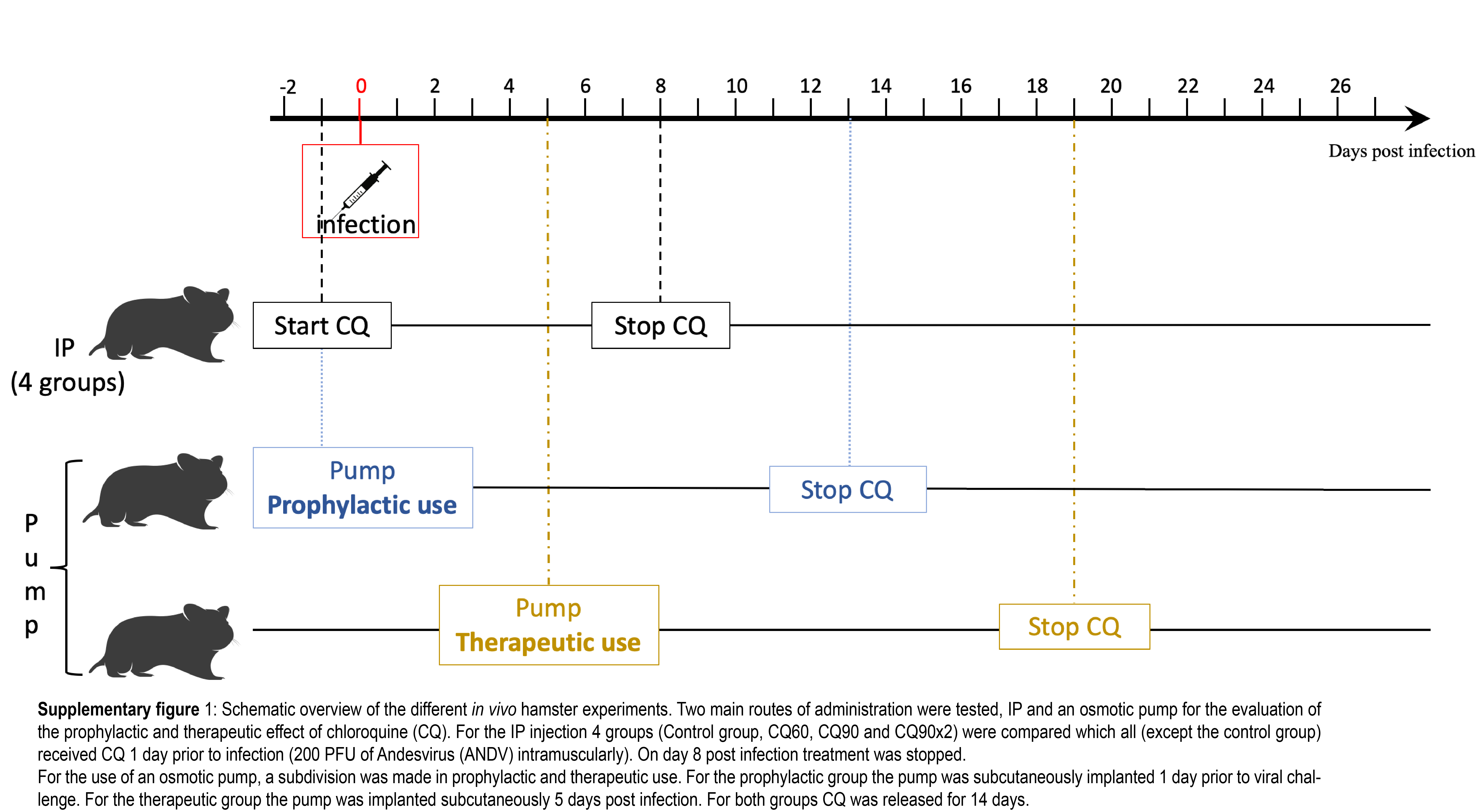

Supplement: Supplementary file 1 [file Image_1.png]

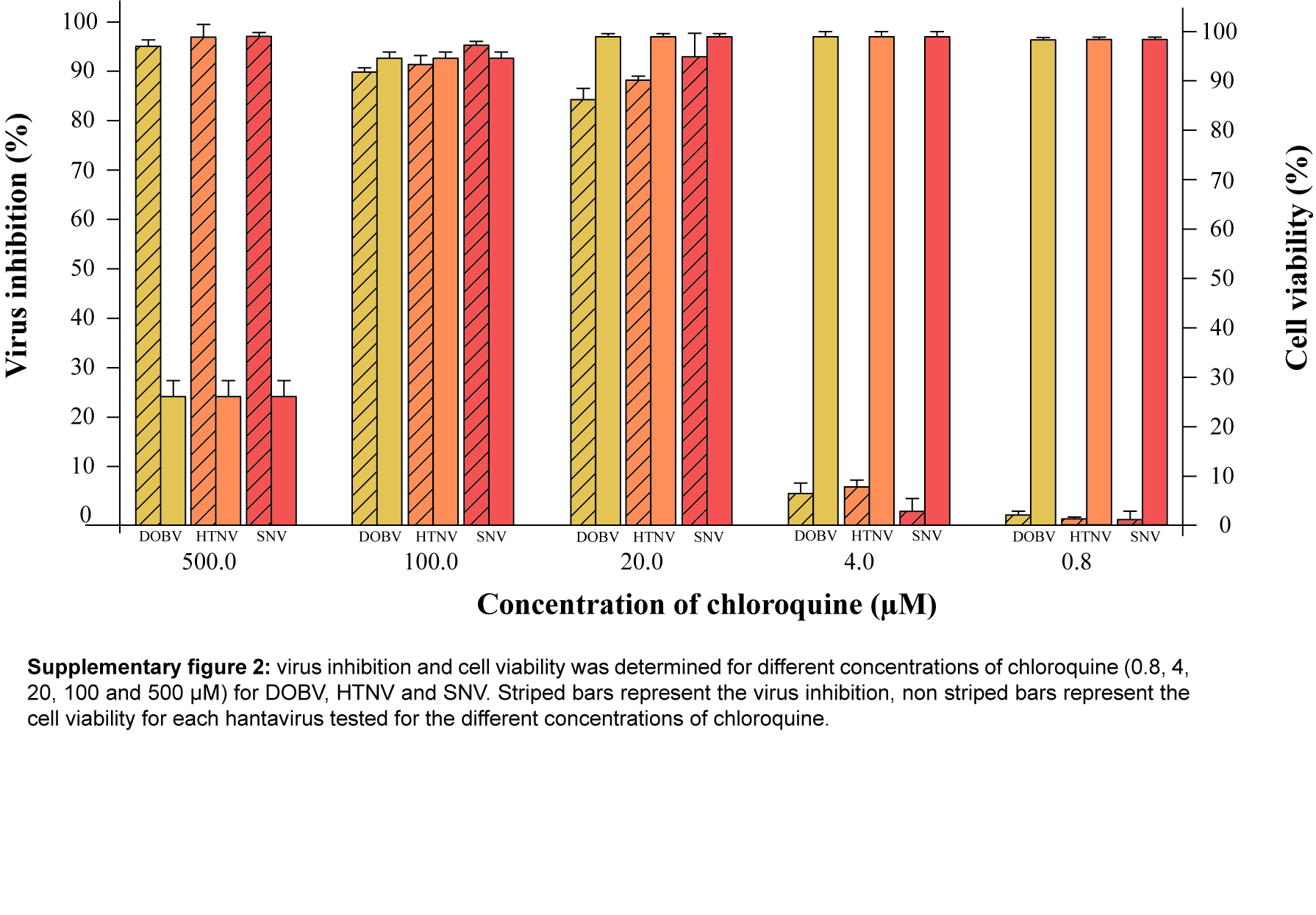

Supplement: Supplementary file 2 [file Image_2.tif]
